# Expecting the unexpected: factors influencing the emergence of fungal and oomycete plant pathogens

**DOI:** 10.1111/nph.16007

**Published:** 2019-07-25

**Authors:** Pilar Corredor‐Moreno, Diane G. O. Saunders

**Affiliations:** ^1^ John Innes Centre Norwich Research Park, Colney Lane Norwich NR4 7UH UK

**Keywords:** climate change, disease surveillance, emergent plant pathogens, host shifts, pathogen pollution, plant pathology

## Abstract

In recent years, the number of emergent plant pathogens (EPPs) has grown substantially, threatening agroecosystem stability and native biodiversity. Contributing factors include, among others, shifts in biogeography, with EPP spread facilitated by the global unification of monocultures in modern agriculture, high volumes of trade in plants and plant products and an increase in sexual recombination within pathogen populations. The unpredictable nature of EPPs as they move into new territories is a situation that has led to sudden and widespread epidemics. Understanding the underlying causes of pathogen emergence is key to managing the impact of EPPs. Here, we review some factors specifically influencing the emergence of oomycete and fungal EPPs, including new introductions through anthropogenic movement, natural dispersal and weather events, as well as genetic factors linked to shifts in host range.

## Introduction

**Figure 1:**
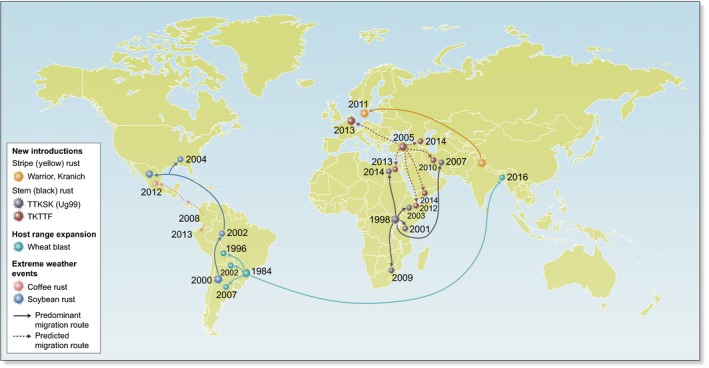
Map illustrating a selection of fungal plant pathogens classified as emergent in recent history. Dates illustrate the first record of a particular pathogen in the given location.

Emergent plant pathogens (EPPs) are plant pathogens that (1) are causal agents of new diseases, (2) display an unusually increased incidence, (3) are able to infect novel hosts, (4) exhibit geographic expansion, and/or (5) have alterations in pathogenesis (Anderson *et al*., 2004). EPPs have caused catastrophic epidemics throughout history and continue to threaten modern agriculture and ecosystem stability (Fig. 1). For instance, the Irish potato famine of the 1840s – the first severe epidemic for which an emerging microorganism was shown as the causal agent (Schumann & D'Arcy, 2000) – caused almost a million deaths in Ireland as a result of starvation and triggered mass migration (Turner, 2005); almost two centuries later, owing to a multitude of factors, the Irish population still has not reached pre‐famine numbers (Yoshida *et al*., 2013). Repeated human introduction of the late blight pathogen *Phytophthora infestans* into nonnative regions was the major cause of potato late blight epidemics. *P. infestans* remains the most important single biotic constraint to potato production world‐wide (Kromann *et al*., 2014) as a result of its high adaptability facilitated by long‐distance dispersal of asexual lineages (Cooke *et al*., 2012; Yoshida *et al*., 2013). Another example of the devastating effects of EPPs is brown spot of rice (*Oryza sativa*), caused by *Cochliobolus miyabeanus*, which was a significant factor in the deaths of over 2 million people during the Bengal famine of 1943 (Sen, 1981).

With the advent of modern resistance gene‐based breeding, regulated use of single‐site fungicides, and improved disease management strategies, it is hoped such catastrophic levels of human suffering can be circumvented in future. However, the timely deployment and ultimate success of such control measures are dependent on a clear understanding of the factors leading to plant disease emergence. Herein, we discuss some main driving factors in emergence, such as new introductions and genetic factors underlying shifts in host range. We focus on information gleaned from oomycete and fungal pathogens, which are responsible for over 30% of all emergent plant diseases (Anderson *et al*., 2004).

## Biogeography of EPPs and the impact of long‐distance dispersal

**Figure 2:**
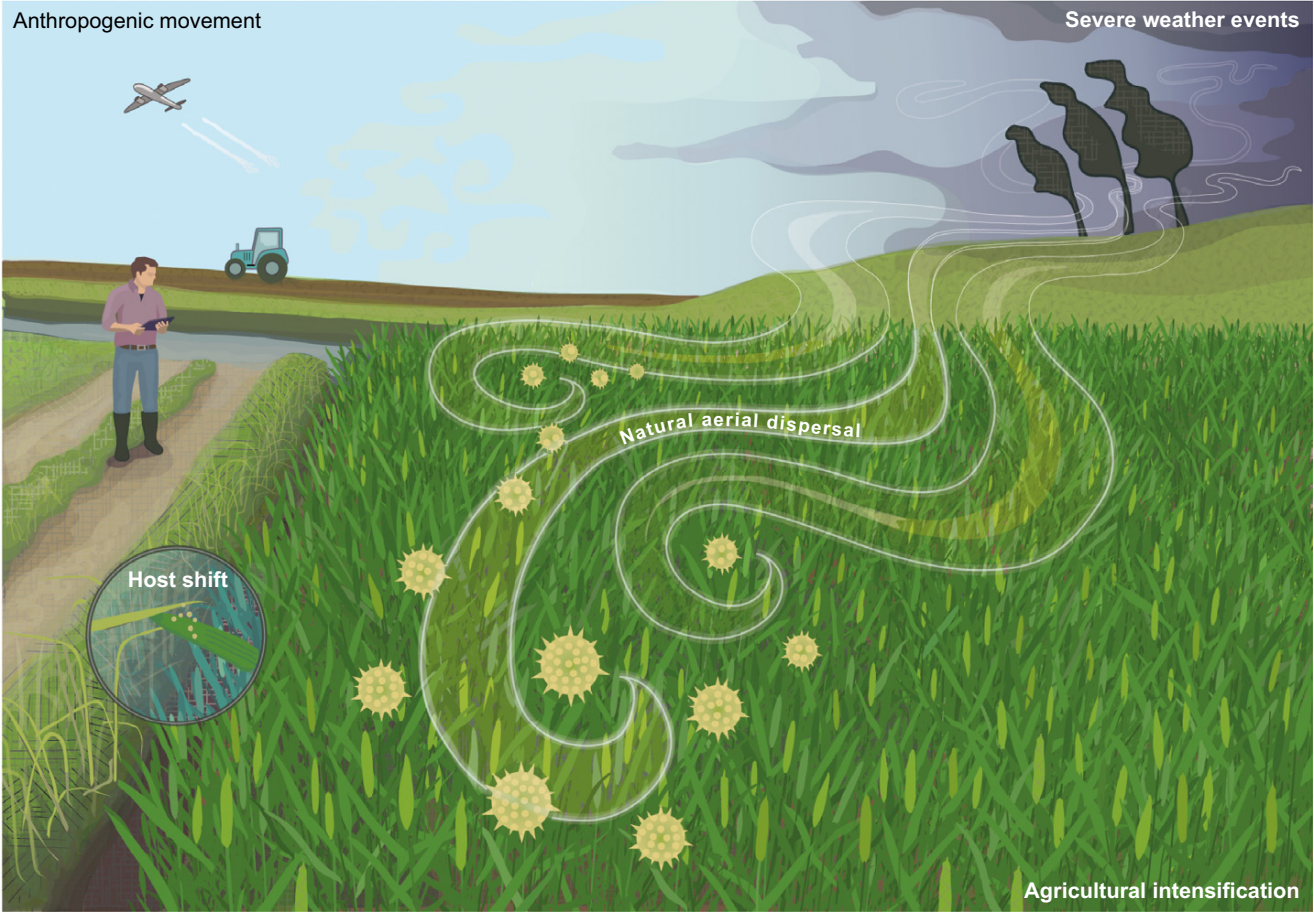
Illustration of the main factors influencing shifts in the biogeography of emergent plant pathogens. The spread of plant pathogens has been facilitated by the global intensification of agriculture, host shifts and new introductions through anthropogenic movement, natural dispersal and severe weather events.

Recent increases in the number of EPPs are largely attributable to alterations in the biogeography of host plants and their pathogens. The spread of plant pathogens in general has been facilitated by high volumes of trade in plants and their products, the global intensification of agriculture with the use of genetically uniform monoculture crops, and perennial crops favouring an increase in sexual recombination in pathogen populations (Anderson *et al*., 2004; Fisher *et al*., 2012). When considering fungal pathogens specifically, two main factors drive emergence: new introductions (including pathogen pollution (Cunningham *et al*., 2003) and natural dispersal) and weather (accounting for *c*. 40% each; Fig. 2) (Anderson *et al*., 2004), alongside alterations in agricultural practices.

### Pathogen pollution

Anthropogenic movement of pathogens is a major cause of EPP introductions. Pathogen pollution, defined as human movement of pathogens outside their natural geographical area or host range (Cunningham *et al*., 2003), enables pathogens to cross geographical boundaries and infect a new host and/or settle in a new region (Anderson *et al*., 2004). For example, the disastrous introduction of the Chestnut blight pathogen *Cryphonectria parasitica* into North America, causing the loss of billions of American chestnut trees, probably occurred via the import of infected saplings from Asia (Dutech *et al*., 2012).

For crop pathogens, one notable example is Karnal bunt, a fungal disease of wheat (*Triticum aestivum*), durum, rye and triticale caused by *Tilletia indica*. The disease was discovered in India in the 1930s and initially restricted to South Asia and Iraq. However, in 1972 it was reported in Mexico, with further isolated outbreaks described in the southwestern United States and South Africa in the late 1990s and early 2000s (Bonde *et al*., 1997). Karnal bunt is a soil‐, seed‐, and air‐borne disease and is probably transmitted primarily via the import of contaminated seed. Increasing concerns have triggered a ban on grain imports from regions with Karnal bunt, with severe economic consequences for the affected countries (Gewin, 2003).

Single‐step incursions are rare, and it is probable that several external invasions are needed for the spread of EPPs into new locations. However, the unprecedented amounts of anthropogenic movement in recent years have clearly facilitated the spread of EPPs into new territories.

### Natural aerial dispersal

Emergent plant pathogens also emerge in new regions via natural dispersal mechanisms. For instance, fungal pathogens that infect above‐ground plant parts frequently disperse via airborne spores, which can assist their entry into new regions. Long‐distance spore dispersal is key for the survival of certain obligate biotrophic fungi, which cannot live on soil or plant debris and must survive in a dormant state or re‐establish the population every season from external sources (Brown & Hovmoller, 2002). Spore dispersal is often essential for re‐establishment, but dispersal is successful only if there is a susceptible host in the target region and favourable environmental conditions. Unfortunately, the reduced genetic diversity of modern crops tends to increase the chances of success of global aerial dispersion of spores (Brown & Hovmoller, 2002) as it only needs one successful incursion to cause widespread damage as a result of crop uniformity.

The recent incursion into western Europe of two races of *Puccinia striiformis* f. sp.* tritici*, the causal agent of wheat yellow rust (Hubbard *et al*., 2015; Hovmøller *et al*., 2016), was attributed to long‐distance spore dispersal from their origin in sexually recombining populations in Asia (Hovmøller *et al*., 2016; Fig. 1). These new races, known as ‘Warrior’ and ‘Kranich’, have unprecedented degrees of genetic diversity and are virulent across many European wheat varieties, posing a serious threat to wheat production (Hubbard *et al*., 2015; Hovmøller *et al*., 2016). Versatility in disease spread assisted by human movement and/or natural wind dispersal can thus enable rapid redistribution of pathogen strains, with dire consequences in regions with high degrees of susceptibility.

### Influence of weather on EPP distribution

Alterations in weather conditions associated with climate change can cause plant pathogens to re‐emerge as significant threats, as dormant pathogens or those previously under control reappear and/or adapt to new geographic regions, leading to severe outbreaks. It has been suggested that milder winters, higher temperatures and higher night temperatures could increase sporulation and infectiousness of foliar fungi (Harvell *et al*., 2002). For example, reductions in the diurnal thermal amplitude decreased the latency period of coffee rust in Central and South America. Together with economic factors, such as the use of susceptible cultivars as a result of high initial investment needed for cultivar replacement, variable rainfall and extreme weather conditions thus led to coffee rust outbreaks in 2008–2013 which directly affected the livelihoods of thousands of smallholder farmers (Avelino *et al*., 2015; Bebber *et al*., 2016) (Fig. 1).

Severe weather events can also influence airborne pathogen dispersal. For instance, hurricane Ivan carried spores of the fungus *Phakopsora pachyrhizi*, the causal agent of Asian soybean rust, into the United States in 2004 from South America, and it is now found throughout the southeastern United States and Mexico (Stokstad, 2004; Fig. 1). Climate change also provides opportunities for the re‐emergence of previously vanquished diseases. It is estimated that crop fungi and oomycetes are moving an average of 6–7 km yr^–1^ poleward (Bebber *et al*., 2013). In western Europe, earlier‐maturing wheat varieties that were bred to prevent the build‐up of wheat stem rust inoculum, which prefers warmer summer temperatures to establish disease, are likely to be at risk. Indeed, in 2013, Germany experienced its first major outbreak of wheat stem rust in decades owing to an unusually cold spring followed by early summer temperatures (Olivera Firpo *et al*., 2017). Stem rust has since been detected in areas of western Europe where it had been absent for many years, the most notable being in Sicily since 2016, where it constituted the largest outbreak in Europe in recent history (Bhattacharya, 2017). Additionally, unusual stem rust outbreaks in Siberia and Northern Kazakhstan in 2015–2017 caused severe yield losses (Hovmøller, 2018). As climate change accelerates, it is difficult to predict which territories EPPs will move to next, and an epidemiological perspective will be crucial to making long‐term predictions about invasion and persistence of EPPs in new areas (Gilligan & van den Bosch, 2008).

### Alteration in agricultural practices

The agricultural landscape contains a very high density of genetically uniform plants that can trigger rapid pathogen cultivar specialization, and facilitate the quick emergence and spread of pathogens (McDonald & Stukenbrock, 2016). Owing to modern agricultural practices, germplasm diversity has reduced globally and high‐yielding cultivars have been specially selected and planted widely for their agronomical value (Van De Wouw *et al*., 2010). This favours the emergence of ‘domesticated’ crop pathogens that evolve quickly and are more virulent than their wild ancestors (McDonald & Stukenbrock, 2016). Large, dense host and pathogen populations also promote coinfection by different pathogens or different strains of the same pathogen, increasing pathogen virulence and the likelihood of horizontal gene transfer.

One example where modern agricultural practices have facilitated pathogen spread is the case of *Zymoseptoria tritici*, the causal agent of *Septoria tritici* Blotch (STB). STB causes significant wheat loses in temperate zones and is the primary wheat foliar disease in most western European countries (Fones & Gurr, 2015). High degrees of host susceptibility have enhanced the population size of *Z. tritici*, promoting substantial gene flow between strains (Zhan & McDonald, 2004). Furthermore, large population sizes coupled with sexual recombination can cause massive shifts in pathogen populations, which can rapidly lead to the evolution of fungicide insensitivity (Stammler & Semar, 2011; Yemelin *et al*., 2017). Accordingly, diversity in the agroecosystem via crop cocultivation, rotation and heterogenous planting of cultivars (McDonald & Stukenbrock, 2016) is crucial to reduce overall pathogen fitness and hinder their ability to rapidly overcome host resistance and fungicide sensitivity.

## Genetic mechanisms underpinning EPP host shifts

**Figure 3:**
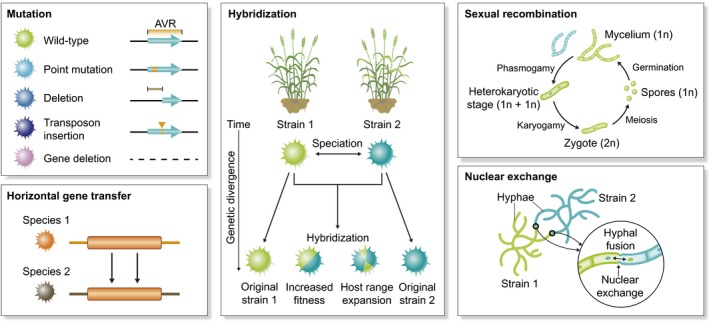
Genetic factors can facilitate host‐range expansion and/or host jumps of fungal or oomycete plant pathogens. Such genetic factors include mutation, horizontal gene transfer, hybridization, sexual recombination and nuclear exchange.

Beyond physical transmission, the emergence of plant pathogens can also be driven by a shift in an existing pathogen's host range. For fungi and oomycetes, host jumps appear to occur even more frequently following anthropogenic movement (Slippers *et al*., 2005). In the agricultural environment, strong selective pressure can aid the pathogen's adaptation to new environmental factors such as host genotypes and ecological conditions. Host‐range expansion and host jumps can be facilitated by genetic events, including mutation, hybridization, sexual recombination and horizontal gene transfer among other mechanisms (Fig. 3).

### Mutation

The most prominent recent example of a host shift followed by migration is the wheat blast pathogen *Magnaporthe oryzae* (syn. *Pyricularia oryzae*) *Triticum* pathotype, which probably emerged from a shift of a *Lolium‐* or *Avena‐*infecting *Pyricularia* population (Ceresini *et al*., 2018). Wheat blast was first detected on wheat in the 1980s in Brazil (Igarashi *et al*., 1986), from where it spread to Bolivia in 1996 and Argentina in 2007 (Ceresini *et al*., 2018). Now well established in South America, it is thought that seedborne inoculum on wheat grain imports from Brazil entered Southeast Asia in 2016, causing severe outbreaks particularly in Bangladesh (Islam *et al*., 2016; Fig. 1). *M. oryzae Triticum* is a diverse group with a broad host range, infecting wheat, barley (*Hordeum vulgare*), rye, and > 10 other grass hosts (Urashima *et al*., 1993; Ceresini *et al*., 2018). With fungicides providing only partial protection and current wheat varieties in Bangladesh displaying limited tolerance at best (CIMMYT; https://www.cimmyt.org/tag/wheat-blast/), wheat blast now poses a serious and immediate threat to wheat production across South Asia.

A key factor in the *M. oryzae* host jump to wheat was loss of recognition induced by mutations or deletions in the *PWT3* and *PWT4* avirulence genes combined with widescale planting of wheat cultivars lacking one or both of the corresponding resistance genes, *Rwt3* and *Rwt4*, that act as a barrier for *Lolium* or *Avena Pyricularia* spp. compatibility on wheat (Inoue *et al*., 2017). As exemplified in this case, when existing host species are physically near novel hosts, the ease of transmission of ‘new’ pathogen strains with adaptive mutations can lead to sudden and widespread epidemics.

### Hybridization and nuclear exchange

Host‐range expansion can also arise as a result of somatic hybridization. For example, nuclear exchange in multinucleated rust fungi in Australia was probably the origin of ‘Scabrum rust’, a hybrid form of the wheat stem rust pathogen (*P. graminis* f. sp. *tritici*) and the rye stem rust pathogen (*P. graminis* f. sp. *secalis*). The three *formae speciales* can all infect barley, causing severe stem rust epidemics in Australia (Luig & Watson, 1972; Park, 2007). The advent of human‐made crops can also provide pathogens with new hosts to exploit. For instance, the wheat–rye hybrid triticale, which had originally been resistant to powdery mildew (Menardo *et al*., 2015), can now be infected by a new *forma specialis* that arose from hybridization between wheat powdery mildew (*B. graminis* f. sp. *tritici*) and rye powdery mildew (*B. graminis* f. sp. *secalis*).

These examples demonstrate the power of hybridization and nuclear exchange as forces in the evolutionary emergence of pathogens. These ‘new’ strains with expanded host range then escape direct competition with their parental strains to exploit their new host niche.

### Sexual recombination and acquisition of accessory chromosomes

Recombination events in filamentous pathogens can enhance virulence and have a direct impact on host‐range expansion (Giraud *et al*., 2010). Many plant pathogens undergo sexual cycles and this is especially notable in agricultural settings, where pathogens with sexual cycles are able to evolve and adapt more readily to changing environments such as new hosts. One example is *Z. tritici*, which has the potential for sexual reproduction at least once per growing season (Cowger *et al*., 2008), leading rapidly to changes in its virulence profile. New species with broader host ranges can also be created by hybrid speciation events, as in the case of *Zymoseptoria pseudotritici*, which has an expanded host range when compared with its wheat‐infecting *Z. tritici* parent (Stukenbrock *et al*., 2012).

Certain fungal species can also exchange ‘pathogenicity’ chromosomes that lead to host‐range expansion. This is certainly the case for the tomato pathogen *Fusarium oxysporum* f. sp. *lycopersici*. The pathogen carries effector genes in one of four accessory chromosomes (Ma *et al*., 2010), which are required for pathogenicity towards its tomato host (Vlaardingerbroek *et al*., 2016). More recently, highly variable fast‐evolving mini‐chromosomes carrying effector sequences have also been identified in the wheat‐adapted lineage of *M. oryzae* (Peng *et al*., 2018). The unstable transmission of these mini‐chromosomes between strains has been hypothesized to act as a mechanism to accelerate host adaptation for *M. oryzae* (Peng *et al*., 2018). From these examples it is clear that sexual recombination and the exchange of accessory chromosomes can play a central role in EPP outbreaks by facilitating the emergence of new pathogen strains with novel virulence combinations that can be crucial for adaptation to new hosts.

### Horizontal gene transfer

Interspecific horizontal gene transfer (HGT) is also a well described mechanism for new plant pathogens to emerge in agricultural settings (Stukenbrock & McDonald, 2008). The acquisition of pathogenicity‐related factors, especially between species coinfecting the same host, can cause rapid emergence of novel pathogens. One notorious example is the transference of the proteinaceous necrotrophic effector ToxA between two wheat fungal pathogens, *Parastagonospora nodorum* and *Pyrenophora tritici‐repentis* (Friesen *et al*., 2006). It is hypothesized that the acquisition of ToxA from *P. nodorum* led to the emergence of a new highly destructive race of *Pyrenophora tritici‐repentis*. Recently, the same host‐specific toxin ToxA was found in *Bipolaris sorokiniana* (McDonald *et al*., 2018), a poorly understood wheat and barley pathogen that causes Helminthosporium leaf blight and common root rot and is the main constraint to cereal production in Central Asia. HGT is a potent driver for pathogen adaptation and in certain cases can have a major role in the emergence of new pathogen races (Stukenbrock & McDonald, 2008). However, for fungal EPPs, the influence of HGT is only just starting to be revealed.

## Increasing preparedness for EPPs

It is crucial that we develop interconnected global surveillance systems to guarantee early warnings and predict pathogen movement within the growing season. Furthermore, understanding the causes of sudden epidemics is key to designing effective control measures. Agricultural practices have led to a ‘domestication’ of plant pathogens. The modern agricultural landscape needs to become more heterogeneous to keep EPPs under control. More regular crop rotation and cultivar turnover, smaller planted areas and an increase in the number of crop species grown per unit area are necessary to reduce the probability of pathogen adaptation and host shifts (McDonald & Stukenbrock, 2016). Accordingly, we need to couple demographic and geographical analyses to population genomic studies to elucidate how plant pathogens evolve and adapt in newly encountered agroecosystems.

### Lessons from the past

Certain plant diseases have been controlled and almost eradicated thanks to effective crop protection strategies such as the use of resistant host cultivars, chemical treatment and/or removal of alternate host plants that form important roles in the life cycle of certain pathogens. For example, in Europe in the late 19^th^ and early 20^th^ centuries, the large‐scale removal of common barberry (*Berberis vulgaris*) ended severe stem rust epidemics in western Europe (Zadoks, 1967). Common barberry is essential for the pathogen (*Puccinia graminis* f.sp.* tritici*) to overwinter in temporal climates and complete sexual reproduction. However, the reintroduction of the alternate host in recent years has the potential to enhance the pathogen's genetic diversity and provide a seasonal haven for stem rust during winter (Smith *et al*., 2009). Accordingly, the first occurrence of a sexual population of stem rust in decades in Sweden was recently reported following repeal of the barberry exclusion law (Berlin, 2017). It is important that such lessons from the past are not forgotten and are instead used to inform and maintain historically effective crop protection strategies.

### Predicting natural EPP dispersal patterns

A useful approach to predicting the dynamics of plant pathogens is to model the behaviour of a given pathogen under known conditions. Mathematical modelling can help to improve surveillance and control systems and possibly predict future outbreaks. Recently, a spore dispersal model for stem rust incorporated international field survey data, global weather data, and a dispersion model to simulate possible disease outbreak scenarios (Meyer *et al*., 2017). Studying the key dispersal routes for stem rust spores, the model predicted when, how often, and how many spores may be dispersed in southern/East Africa, the Middle East, and Central/South Asia for highly virulent strains such as Ug99 (Pretorius *et al*., 2000; Meyer *et al*., 2017). Such results can highlight potential dispersion routes and guide cost‐effective control strategies.

Incorporating knowledge from population genomics into disease modelling should also increase our ability to predict the evolutionary route of plant pathogens in the agricultural landscape. For wheat blast, planting wheat varieties with the *Rwt3* and *Rwt4* resistance genes should limit *Lolium* or *Avena Pyricularia* spp. from further jumping the barrier to wheat, as a result of the presence of the corresponding *PWT3* and *PWT4* avirulence genes (Inoue *et al*., 2017). Thus, a clear understanding of how plant pathogen genomes evolve should be incorporated into dispersal models to improve their predictive ability and value for EPP prevention.

### Interconnected disease surveillance systems

One example of an effective national network for disease surveillance is the National Plant Diagnostic Network, which focuses on early detection, accurate diagnosis, and rapid communication to help control the spread of EPPs within the United States (National Plant Diagnostic Network, 2019). Such rigorous systems provide a valuable tool to manage local outbreaks; however, as pathogens shift location, EPP surveillance and reporting need international alliances to succeed. For example, the ‘Digalu’ (TKTTF) race of stem rust was initially identified in Turkey in 2007–2008 with minimal losses (Mert *et al*., 2012); however, once it entered Ethiopia in 2013, the race caused near‐total field losses as a result of the high degree of susceptibility of the widely grown wheat cv ‘Digalu’ (Olivera *et al*., 2015; Fig. 1). Interconnected screening networks testing dominant pathogen races across global germplasm collections could be used to circumvent similar outbreaks in the future.

Since 1993, the international standards for phytosanitary measures dictated by the Food and Agriculture Organisation (FAO) of the United Nations have required countries to report plant pests and diseases to the International Plant Protection Convention (FAO, 2006; International Plant Protection Convention, 2012), which provides a framework for pathogen monitoring, surveillance and management. However, the achievement of accuracy in these reports can be hampered by economic consequences, political barriers and a lack of standardization in testing procedures. Given the global reach and ease of transmission of EPPs, it is essential we develop a unified and transparent international strategy to support the accurate and timely reporting of EPPs (Carvajal‐Yepes *et al*., 2019).

## Conclusions

As human activities continue to erode natural barriers to dispersal, human‐mediated pathogen redistribution poses an increasing threat to the stability of agricultural systems and native biodiversity (Kolar & Lodge, 2001). The versatility EPPs display in their spread emphasises the urgent need for a coordinated globalisation of disease monitoring systems to develop proactive management strategies for EPPs at high risk of movement into vulnerable areas. Fortunately, modern agricultural practices such as resistance breeding and fungicides provide us with better protection than during the catastrophic EPP outbreaks that have plagued history. However, we must remain vigilant to the threats posed by EPPs to plant health and global food security, in order to safeguard and maximise crop production as productivity demands continue to increase in the future.

## References

[nph16007-bib-0001] Anderson PK , Cunningham AA , Patel NG , Morales FJ , Epstein PR , Daszak P . 2004 Emerging infectious diseases of plants: pathogen pollution, climate change and agrotechnology drivers. Trends in Ecology and Evolution 19: 535–544. 16701319 10.1016/j.tree.2004.07.021

[nph16007-bib-0002] Avelino J , Cristancho M , Georgiou S , Imbach P , Aguilar L , Bornemann G , Läderach P , Anzueto F , Hruska AJ , Morales C . 2015 The coffee rust crises in Colombia and Central America (2008–2013): impacts, plausible causes and proposed solutions. Food Security 7: 303–321.

[nph16007-bib-0003] Bebber DP , Castillo AD , Gurr SJ . 2016 Modelling coffee leaf rust risk in Colombia with climate reanalysis data. Philosophical Transactions of the Royal Society of London. Series B: Biological Sciences 371: 20150458. 28080984 10.1098/rstb.2015.0458 PMC5095537

[nph16007-bib-0004] Bebber DP , Ramotowski MAT , Gurr SJ . 2013 Crop pests and pathogens move polewards in a warming world. Nature Climate Change 3: 985–988.

[nph16007-bib-0005] Berlin A . 2017 Stem rust attacks in Sweden heralds the return of a previously vanquished foe. Sweden: Swedish University of Agricultural Sciences, SLU News.

[nph16007-bib-0006] Bhattacharya S . 2017 Deadly new wheat disease threatens Europe's crops. Nature 542: 145–146. 28179687 10.1038/nature.2017.21424

[nph16007-bib-0007] Bonde MR , Peterson GL , Schaad NW , Smilanick JL . 1997 Karnal bunt of wheat. Plant Disease 81: 1370–1377. 30861787 10.1094/PDIS.1997.81.12.1370

[nph16007-bib-0008] Brown JK , Hovmoller MS . 2002 Aerial dispersal of pathogens on the global and continental scales and its impact on plant disease. Science 297: 537–541. 12142520 10.1126/science.1072678

[nph16007-bib-0009] Carvajal‐Yepes M , Cardwell K , Nelson A , Garrett KA , Giovani D , Saunders DGO , Kamoun S , Legg JP , Verdier V , Lessel J , *et al* 2019 A global surveillance system for crop diseases. Science 364: 1237–1239. 31249049 10.1126/science.aaw1572

[nph16007-bib-0010] Ceresini PC , Lili V , Rios JA , Aucique‐p CE , Moreira SI , Alves E , Croll D , Maciel N . 2018 Wheat blast: past, present, and future. Annual Review of Phytopathology 56: 427–456. 10.1146/annurev-phyto-080417-050036 29975608

[nph16007-bib-0012] Cooke DEL , Cano LM , Raffaele S , Bain RA , Cooke LR , Etherington GJ , Deahl KL , Farrer RA , Gilroy EM , Goss EM , *et al* 2012 Genome analyses of an aggressive and invasive lineage of the Irish potato famine pathogen. PLoS Pathogens 8: e1002940. 23055926 10.1371/journal.ppat.1002940 PMC3464212

[nph16007-bib-0013] Cowger C , Brunner PC , Mundt CC . 2008 Frequency of sexual recombination by *Mycosphaerella graminicola* in mild and severe epidemics. Phytopathology 98: 752–759. 18943250 10.1094/PHYTO-98-7-0752

[nph16007-bib-0014] Cunningham AA , Daszak P , Rodríguez JP . 2003 Pathogen pollution: defining a parasitlogical threat to biodiversity conservation. Journal of Parasitology 89: S78–S83.

[nph16007-bib-0015] Dutech C , Barrès B , Bridier J , Robin C , Milgroom MG , Ravignè V . 2012 The chestnut blight fungus world tour: successive introduction events from diverse origins in an invasive plant fungal pathogen. Molecular Ecology 21: 3931–3946. 22548317 10.1111/j.1365-294X.2012.05575.x

[nph16007-bib-0016] FAO . 2006 International standards for phytosanitary measures. [WWW document] URL http://www.fao.org/3/a0450e/a0450e.pdf [accessed 9 June, 2019].

[nph16007-bib-0017] Fisher MC , Henk DA , Briggs CJ , Brownstein JS , Madoff LC , McCraw SL , Gurr SJ . 2012 Emerging fungal threats to animal, plant and ecosystem health. Nature 484: 186–194. 22498624 10.1038/nature10947 PMC3821985

[nph16007-bib-0018] Fones H , Gurr S . 2015 The impact of *Septoria tritici* Blotch disease on wheat: an EU perspective. Fungal Genetics and Biology 79: 3–7. 26092782 10.1016/j.fgb.2015.04.004 PMC4502551

[nph16007-bib-0019] Friesen TL , Stukenbrock EH , Liu Z , Meinhardt S , Ling H , Faris JD , Rasmussen JB , Solomon PS , McDonald BA , Oliver RP . 2006 Emergence of a new disease as a result of interspecific virulence gene transfer. Nature Genetics 38: 953–956. 16832356 10.1038/ng1839

[nph16007-bib-0020] Gewin V . 2003 Bioterrorism: agriculture shock. Nature 421: 106–108. 12520273 10.1038/421106a

[nph16007-bib-0021] Gilligan CA , van den Bosch F . 2008 Epidemiological models for invasion and persistence of pathogens. Annual Review of Phytopathology 46: 385–418. 10.1146/annurev.phyto.45.062806.094357 18680429

[nph16007-bib-0022] Giraud T , Gladieux P , Gavrilets S . 2010 Linking the emergence of fungal plant diseases with ecological speciation. Trends in Ecology and Evolution 25: 387–395. 20434790 10.1016/j.tree.2010.03.006 PMC2885483

[nph16007-bib-0023] Harvell CD , Mitchell CE , Ward JR , Altizer S , Dobson AP , Ostfeld RS , Samuel MD . 2002 Climate warming and disease risks for terrestrial and marine biota. Science 296: 2158–2162. 12077394 10.1126/science.1063699

[nph16007-bib-0024] Hovmøller MS . 2018 Global rust reference center. [WWW document] URL http://wheatrust.org/. [accessed 5 November 2018].

[nph16007-bib-0025] Hovmøller MS , Walter S , Bayles RA , Hubbard A , Flath K , Sommerfeldt N , Leconte M , Czembor P , Rodriguez‐Algaba J , Thach T *et al* 2016 Replacement of the European wheat yellow rust population by new races from the centre of diversity in the near‐Himalayan region. Plant Pathology 65: 402–411.

[nph16007-bib-0026] Hubbard A , Lewis CM , Yoshida K , Ramirez‐Gonzalez RH , de Vallavieille‐Pope C , Thomas J , Kamoun S , Bayles R , Uauy C , Saunders DG . 2015 Field pathogenomics reveals the emergence of a diverse wheat yellow rust population. Genome Biology 16: 23. 25723868 10.1186/s13059-015-0590-8 PMC4342793

[nph16007-bib-0027] Igarashi S , Utiamada C , Igarashi L , Kazuma A , Lopes R . 1986 Ocurrence of *Pyrcularia* sp. in wheat (*Triticum aestivum* L.) in the State of Parana, Brazil. Fitopatologia Brasileira 36: 79–82.

[nph16007-bib-0028] Inoue Y , Vy TTP , Yoshida K , Asano H , Mitsuoka C , Asuke S , Anh VL , Cumagun CJR , Chuma I , Terauchi R *et al* 2017 Evolution of the wheat blast fungus through functional losses in a host specificity determinant. Science 357: 80–83. 28684523 10.1126/science.aam9654

[nph16007-bib-0029] International Plant Protection Convention . 2012 IPPC strategic framework. [WWW document] URL https://www.ippc.int/static/media/files/publications/en/2013/06/03/1344410402_ippc_strategicframework_e_w_201305101054en.pdf. [accessed 9 June 2019].

[nph16007-bib-0030] Islam MT , Croll D , Gladieux P , Soanes DM , Persoons A , Bhattacharjee P , Hossain MS , Gupta DR , Rahman MM , Mahboob MG *et al* 2016 Emergence of wheat blast in Bangladesh was caused by a South American lineage of *Magnaporthe oryzae* . BMC Biology 14: 84. 27716181 10.1186/s12915-016-0309-7 PMC5047043

[nph16007-bib-0031] Kolar CS , Lodge DM . 2001 Progress in invasion biology: predicting invaders. Trends in Ecology and Evolution 16: 199–204. 11245943 10.1016/s0169-5347(01)02101-2

[nph16007-bib-0032] Kromann P , Miethbauer T , Ortiz O , Forbes GA . 2014 Review of potato biotic constraints and experiences with integrated pest management interventions In: Pimentel D, Peshin R, eds. Integrated pest management. Dordrecht, the Netherlands: Springer, 245–268.

[nph16007-bib-0033] Luig NH , Watson IA . 1972 The role of wild and cultivated grasses in the hybridization of formae speciales of *Puccinia graminis* . Australian Journal of Biological Sciences 25: 335–342.

[nph16007-bib-0034] Ma LJ , Van Der Does HC , Borkovich KA , Coleman JJ , Daboussi MJ , Di Pietro A , Dufresne M , Freitag M , Grabherr M , Henrissat B *et al* 2010 Comparative genomics reveals mobile pathogenicity chromosomes in *Fusarium* . Nature 464: 367–373. 20237561 10.1038/nature08850 PMC3048781

[nph16007-bib-0036] McDonald BA , Stukenbrock EH . 2016 Rapid emergence of pathogens in agro‐ecosystems: global threats to agricultural sustainability and food security. Philosophical Transactions of the Royal Society of London. Series B: Biological Sciences 371: 20160026. 28080995 10.1098/rstb.2016.0026 PMC5095548

[nph16007-bib-0035] McDonald MC , Ahren D , Simpfendorfer S , Milgate A , Solomon PS . 2018 The discovery of the virulence gene ToxA in the wheat and barley pathogen *Bipolaris sorokiniana* . Molecular Plant Pathology 19: 432–439. 28093843 10.1111/mpp.12535 PMC6638140

[nph16007-bib-0037] Menardo F , Praz C , Wyder S , Bourras SA , McNally KE , Parlange F , Riba A , Roffler S , Schaefer L , Shimizu KK *et al* 2015 Hybridization of powdery mildew strains gives raise to pathogens on novel agricultural crop species. Nature Genetics 48: 201–205. 10.1038/ng.3485 26752267

[nph16007-bib-0038] Mert Z , Karakaya A , Düşünceli F , Akan K , Çetin L . 2012 Determination of *Puccinia graminis f. sp. tritici* races of wheat in Turkey. Turkish Journal of Agriculture and Forestry 36: 107–120.

[nph16007-bib-0039] Meyer M , Cox JA , Hitchings MDT , Burgin L , Hort MC , Hodson DP , Gilligan CA . 2017 Quantifying airborne dispersal routes of pathogens over continents to safeguard global wheat supply. Nature Plants 3: 780–786. 28947769 10.1038/s41477-017-0017-5

[nph16007-bib-0040] National Plant Diagnostic Network . 2019 National plant diagnostic network website [WWW document]. URL https://www.npdn.org/home [accessed 5 November 2018].

[nph16007-bib-0041] Olivera Firpo PD , Newcomb M , Flath K , Sommerfeldt‐Impe N , Szabo LJ , Carter M , Luster DG , Jin Y . 2017 Characterization of *Puccinia graminis* f. sp. *tritici* isolates derived from an unusual wheat stem rust outbreak in Germany in 2013. Plant Pathology 66: 1258–1266.

[nph16007-bib-0042] Olivera P , Newcomb M , Szabo LJ , Rouse M , Johnson J , Gale S , Luster DG , Hodson D , Cox JA , Burgin L *et al* 2015 Phenotypic and genotypic characterization of race TKTTF of *Puccinia graminis* f. sp. *tritici* that caused a wheat stem rust epidemic in southern Ethiopia in 2013–14. Phytopathology 105: 917–928. 25775107 10.1094/PHYTO-11-14-0302-FI

[nph16007-bib-0043] Park RF . 2007 Stem rust of wheat in Australia. Australian Journal of Agricultural Research 58: 558–566.

[nph16007-bib-0044] Peng Z , Garcia EO , Lin G , Hu Y , Dalby M , Migeon P , Tang H , Farman M , Cook D , White FF *et al* 2018 Effector gene reshuffling involves dispensable mini‐chromosomes in the wheat blast fungus. bioRxiv. doi: http://10.1101/359455. 10.1371/journal.pgen.1008272 PMC6741851 31513573

[nph16007-bib-0045] Pretorius ZA , Singh RP , Wagoire WW , Payne TS . 2000 Detection of virulence to wheat stem rust resistance gene Sr31 in *Puccinia graminis*. f. sp. *tritici* in Uganda. Plant Disease 84: 203.2. 10.1094/PDIS.2000.84.2.203B 30841334

[nph16007-bib-0046] Schumann GL , D'Arcy CJ . 2000 Late blight of potato and tomato. *The Plant Health Instructor;* doi: 10.1094/phi-i-2000-0724-01.

[nph16007-bib-0047] Sen A . 1981 The great Bengal famine In: Poverty and Famines: an essay on entitlement and deprivation. Oxford, UK: Oxford University Press, 52–85.

[nph16007-bib-0048] Slippers B , Stenlid J , Wingfield MJ . 2005 Emerging pathogens: fungal host jumps following anthropogenic introduction. Trends in Ecology and Evolution 20: 420–421. 16701409 10.1016/j.tree.2005.05.002

[nph16007-bib-0049] Smith K , Draper M , Simmons K , Bennett R , Hebbar P , Royer M , Murray T . 2009 US preparations for potential introduction of Ug99 strains of wheat stem rust. Outlooks on Pest Management 20: 148–152.

[nph16007-bib-0050] Stammler G , Semar M . 2011 Sensitivity of *Mycosphaerella graminicola* (anamorph: *Septoria tritici*) to DMI fungicides across Europe and impact on field performance. EPPO Bulletin 41: 149–155.

[nph16007-bib-0051] Stokstad E . 2004 Plant pathologists gear up for battle with dread fungus. Science 306: 1672–1673. 15576584 10.1126/science.306.5702.1672

[nph16007-bib-0052] Stukenbrock EH , Christiansen FB , Hansen TT , Dutheil JY , Schierup MH . 2012 Fusion of two divergent fungal individuals led to the recent emergence of a unique widespread pathogen species. Proceedings of the National Academy of Sciences, USA 109: 10954–10959. 10.1073/pnas.1201403109 PMC3390827 22711811

[nph16007-bib-0053] Stukenbrock EH , McDonald BA . 2008 The origins of plant pathogens in agro‐ecosystems. Annual Review of Phytopathology 46: 75–100. 10.1146/annurev.phyto.010708.154114 18680424

[nph16007-bib-0054] Turner RS . 2005 After the famine: plant pathology, *Phytophthora infestans*, and the late blight of potatoes, 1845–1960. Historical Studies in the Physical and Biological Sciences 35: 341–370.

[nph16007-bib-0055] Urashima AS , Igarashi S , Kato H . 1993 Host range, mating type, and fertility of *Pyricularia grisea* from wheat in Brazil. Plant Disease 77: 1211–1216.

[nph16007-bib-0056] Van De Wouw M , Kik C , Van Hintum T , Van Treuren R , Visser B . 2010 Genetic erosion in crops: concept, research results and challenges. Plant Genetic Resources: Characterisation and Utilisation 8: 1–15.

[nph16007-bib-0057] Vlaardingerbroek I , Beerens B , Schmidt SM , Cornelissen BJC , Rep M . 2016 Dispensable chromosomes in *Fusarium oxysporum* f. sp. *lycopersici* . Molecular Plant Pathology 17: 1455–1466. 27271322 10.1111/mpp.12440 PMC6638487

[nph16007-bib-0058] Yemelin A , Brauchler A , Jacob S , Laufer J , Heck L , Foster AJ , Antelo L , Andresen K , Thines E . 2017 Identification of factors involved in dimorphism and pathogenicity of *Zymoseptoria tritici* . PLoS ONE 12: e0183065. 28829795 10.1371/journal.pone.0183065 PMC5568738

[nph16007-bib-0059] Yoshida K , Schuenemann VJ , Cano LM , Pais M , Mishra B , Sharma R , Lanz C , Martin FN , Kamoun S , Krause J *et al* 2013 The rise and fall of the Phytophthora infestans lineage that triggered the Irish potato famine. eLife 2: e00731. 23741619 10.7554/eLife.00731 PMC3667578

[nph16007-bib-0060] Zadoks J . 1967 Epidemiology of wheat rusts in Europe. Pest Articles & News Summaries Section B. Plant Disease Control 13: 29–46.

[nph16007-bib-0061] Zhan J , McDonald BA . 2004 The interaction among evolutionary forces in the pathogenic fungus *Mycosphaerella graminicola* . Fungal Genetics and Biology 4: 590–599. 10.1016/j.fgb.2004.01.006 15121082

